# The influence of maternal gestational weight gain on adverse perinatal outcomes

**DOI:** 10.3389/fendo.2025.1513344

**Published:** 2025-02-05

**Authors:** Qingshan Yan, Wenya Cai, Yong Guo

**Affiliations:** ^1^ Department of Health Care, Guangdong Women and Children Hospital, Guangzhou, China; ^2^ Department of Basic Medicine and Public Health, Jinan University, Guangzhou, China; ^3^ Department of Public Health, Guangzhou Medical University, Guangzhou, China

**Keywords:** gestational weight gain, gestational hypertension, birth outcome, pregnancy, large-for-gestational-age

## Abstract

**Objective:**

To analyze the impact of maternal gestational weight gain (GWG) on adverse outcomes for mothers and infants.

**Methods:**

A retrospective analysis was conducted, collecting relevant information on 9,058 singleton pregnancies and newborns from prenatal check-ups and deliveries at Guangdong Women and Children Hospital from 2017 to 2022. The subjects were grouped according to different GWG, and logistic regression was used to analyze the impact of GWG on adverse outcomes, including hypertensive disorders of pregnancy (HDP), gestational diabetes mellitus (GDM), large-for-gestational-age infants (LGA), small-for-gestational-age infants (SGA), and preterm births.

**Results:**

Among the 9058 participants included in the study, there were 438 cases (4.8%) of HDP, including 266 cases (2.9%) of gestational hypertension and 172 cases (1.9%) of preeclampsia. Additionally, there were 2018 cases (22.3%) of GDM; Among the adverse birth outcomes for newborns, the prevalence was 9.7% for SGA, 9.9% for LGA, 1.4% for early/mid-term preterm births, and 4.2% for late preterm births. After adjusting for confounding factors, the results showed that, excessive GWG was a risk factor for HDP (OR=1.829, *P*<0.05) and LGA (OR=1.792, P<0.05) compared to the normal gestational weight gain group. Insufficient GWG increased the risk of GDM (OR=2.203, P<0.05), SGA (OR=1.474, P<0.05) and was also a risk factor for early/mid-term preterm infants (OR=3.326, P<0.05) and late preterm infants (OR=1.715, P<0.05).

**Conclusion:**

Excessive GWG is a risk factor for the occurrence of HDP and LGA, while insufficient GWG increases the risk of GDM, SGA and preterm infants (especially early/mid-term preterm infants). Therefore, it is recommended to strengthen the monitoring of weight changes during pregnancy in women of childbearing age, guide nutritional management during pregnancy, and keep GWG within a reasonable range to prevent adverse outcomes for mothers and infants.

## 1 Introduction

Gestational weight gain (GWG) is a cumulative measure of the changing physiology of the mother (fat-free and fat mass accumulation, blood volume expansion), placental weight, and the developing fetus (fat- and fat-free mass as well as the amniotic fluid accretion) (1). It is an essential condition for ensuring fetal health; however, insufficient or excessive GWG can lead to various adverse outcomes. For instance, pregnant women with inadequate weight gain face increased risks of anemia, preterm birth, and delivering small for gestational age (SGA) neonates, while those with excessive GWG are more likely to experience hypertensive disorders of pregnancy (HDP), gestational diabetes mellitus (GDM), and the delivery of large for gestational age (LGA) (2–4), furthermore, excessive GWG may indirectly influence the probability of obesity in offspring during childhood (5, 6).

Currently, the incidence of various types of adverse outcomes for mothers and infants remains high, with epidemiologic surveys showing that in the United States, the prevalence of hypertension in pregnancy had increased from 2.79% in 1989 to 8.22% in 2020 (7); A retrospective study conducted across 18 medical centers in 12 provinces of China indicated that the overall prevalence of macrosomia in these provinces reached 7.3% in 2018 (8). As a significant factor influencing the occurrence of these adverse outcomes, GWG is worth to close attention and monitoring. In 2022, China established recommended standards for GWG, incorporating pre-pregnancy body mass index (BMI) and defining appropriate GWG ranges based on different pre-pregnancy BMI categories (9). This standard has been shown to be more suitable for pregnant women in China than the GWG recommendations set by the Institute of Medicine in 2009 (10, 11).This study, based on the established standards, investigated the maternal and infant outcomes associated with varying GWG among pregnant women who delivered at Guangdong Women and Children Hospital from 2017 to 2022, providing a theoretical basis for better guidance on appropriate weight gain (AGA) during pregnancy in the future.

## 2 Materials and methods

### 2.1 Study population

This study employed a retrospective analysis methodology, utilizing the electronic medical record system of Guangdong Women and Children Hospital to collect relevant information from all mothers who delivered there between 2017 and 2022.

Inclusion criteria for the study: completed inpatient medical records; singleton live birth; no pre-existing chronic diseases prior to pregnancy, such as hypertension, diabetes, or immune system disorders. Exclusion criteria: multiple pregnancies; still birth; fetal chromosomal abnormalities; fetal malformations; missing key medical data such as gestational age and birth weight. This study addressed missing, duplicated, and anomalous data samples, ultimately including 9,058 subjects in accordance with the established exclusion criteria.

### 2.2 Data collection

The data for this study was sourced from the electronic medical record system of Guangdong Women and Children Hospital. It includes the collection of maternal coding, date of birth, height, pre-pregnancy weight, weight at delivery, late pregnancy systolic and diastolic blood pressure, gestational age, admission and discharge diagnoses, as well as neonatal data such as date of birth, birthweight, sex, and mode of delivery for mothers who delivered at the hospital from 2017 to 2022.

### 2.3 The classification of research indicators

BMI is calculated using the formula: BMI= weight (kg)/height² (m²). According to the data analysis by the China Obesity Working Group, BMI is categorized into four groups: underweight (BMI < 18.5 kg/m²), normal weight (18.5 kg/m² ≤ BMI < 24.0 kg/m²), overweight (24.0 kg/m² ≤ BMI < 28.0 kg/m²), and obesity (BMI ≥ 28.0 kg/m²) (12). The pre-pregnancy weight and height are recorded values from the mother’s early pregnancy or pre-pregnancy documentation.

GWG is defined as the difference between the weight at delivery and the weight measured in the first trimester. According to the Standard of Recommendation for Weight Gain during Pregnancy Period which have been issued for Chinese women in 2022 (9), the normal range for GWG is as follows: 11.0–16.0 kg for underweight pre-pregnancy weight, 8.0–14.0 kg for normal pre-pregnancy weight, 7.0–11.0 kg for overweight pre-pregnancy weight, and 5.0–9.0 kg for obese pre-pregnancy weight. GWG is classified into three groups: those below the lower limit of the range are considered insufficient GWG, those above the upper limit are considered excessive GWG, and those within the range are considered normal GWG.

### 2.4 Definition of outcome

In our study, HDP include gestational hypertension, mild preeclampsia, and severe preeclampsia. Due to the relatively small number of cases of mild and severe preeclampsia, they were combined for analysis. Among them, gestational hypertension was defined as new-onset hypertension with resting blood pressure ≥140/90 mmHg after the 20th gestational week in previously normotensive women, with negative proteinuria, and normalization of blood pressure within 12 weeks postpartum; Gestational hypertension accompanied by other clinical manifestations of preeclampsia, such as positive proteinuria or involvement of other organs or systems, was diagnosed as preeclampsia (13). In this study, these diagnoses were all obtained from medical record documentations.

A 75g oral glucose tolerance test (OGTT) was conducted, and GDM was diagnosed if any of the following criteria were met: fasting blood glucose ≥5.1 mmol/L, 1-hour blood glucose ≥10.0 mmol/L, or 2-hour blood glucose ≥8.5 mmol/L (14). The diagnoses for GDM in this study were based on the medical records.

According to the growth assessment standards for newborns at different gestational ages released by the National Health Commission of China in 2022, newborns are categorized based on their birthweight. By considering both the birth weight and gestational age, newborns are classified into three groups: SGA, AGA, and LGA. In this classification, SGA refers to newborns whose birth weight is below the 10th percentile of the average weight for their gestational age. AGA indicates newborns whose birth weight falls between the 10th and 90th percentiles of the average weight for their gestational age. LGA describes newborns whose birth weight is above the 90th percentile of the average weight for their gestational age.

According to gestational age, newborns are classified as follows: early/mid-term Preterm Infants: early preterm infants (gestational age < 32 weeks) and mid-term preterm infants (gestational age between 32 and 33 weeks); late Preterm Infants: gestational age between 34 and 36 weeks; term infants: infants born at term (gestational age ≥ 37 weeks).

### 2.5 Statistical analysis

This study employed SPSS.26 for statistical analysis of the data. Descriptive analysis of research variables was performed according to three groups of GWG. For the normally distributed data, we used $\bar{x}\pm s$ deviation for description, and for the skewed distributed data, we used median (IQR). And inter-group comparisons conducted using t-tests. Qualitative variables were represented by frequency and percentage (%) and compared between groups using the *χ* ^2^ test. After adjusting for potential confounding factors such as age, pre-pregnancy BMI, and newborn sex, a logistic regression model was used to analyze the association between different GWG classifications and outcome variables (HDP, GDM, LGA, SGA and preterm infants), calculating the adjusted odds ratios (aOR) and 95% confidence intervals (CI). A p-value of <0.05 was considered statistically significant.

## 3 Result

### 3.1 Participants’ characteristics

A total of 9,058 women were included in this study, with an average age of 36.0 ± 4.93 years. The average pre-pregnancy BMI was 20.8 ± 2.68 kg/m², with 1787 women (19.7%) categorized as underweight, 6122 women (67.6%) as normal weight, 1030 women (11.4%) as overweight, and 119 women (1.3%) as obese. The average admission blood pressure was 117 ± 11.7 mmHg for systolic pressure and 71.2 ± 8.89 mmHg for diastolic pressure. The number of vaginal deliveries was 5515 (60.9%), which was more than the number of cesarean deliveries; of the newborns, 4161 (45.9%) were girls and 4897 (54.1%) were boys. The general characteristics of the mothers were statistically significant (P<0.05) when comparing the weight gained during the different trimesters.

In adverse maternal and neonatal outcomes, among the mothers, there were 438 cases (4.8%) of HDP, including 266 cases (2.9%) of gestational hypertension, and 172 cases (1.9%) of preeclampsia. Compared with mothers with normal or insufficient GWG, those with excessive GWG had a significantly higher likelihood of developing HDP (P <0.05). In addition, there were 2018 cases (22.3%) of GDM. Regarding neonatal birthweight, there were 877 cases (9.7%) of SGA and 897 cases (9.9%) of LGA. The incidence of SGA was significantly higher in the insufficient GWG group compared to the other two groups (P < 0.05), while the incidence of LGA was greater in the excessive GWG group (P < 0.05). Furthermore, there were 123 cases (1.4%) of early/mid-term preterm infants and 381 cases (4.2%) of late preterm infants, with significant statistical differences observed among the three groups (**Table 1**).

**Table 1:** General clinical characteristics of study subjects in different GWG groups.

	GWG category			All (N=9058)	P-value
	Inadequate (N=1048)	Adequate (N=4127)	Excessive (N=3883)		
Age	36.4 ± 5.10	36.2 ± 4.92	35.8 ± 4.88	36.0 ± 4.93	<0.05
<25	1 (0.1)	11 (0.3)	11 (0.3)	23 (0.3)	0.031
25~<35	412 (39.3)	1602 (38.8)	1654 (42.6)	3668 (40.5)	
≥35	635 (60.6)	2514 (60.9)	2218 (57.1)	5367 (59.3)	
Pre-pregnancy BMI category	21.1 ± 3.17	20.5 ± 2.53	21.0 ± 2.66	20.8 ± 2.68	<0.05
Underweight	308 (29.4)	939 (22.8)	540 (13.9)	1787 (19.7)	<0.05
Normal weight	539 (51.4)	2851 (69.1)	2732 (70.4)	6122 (67.6)	
Overweight	181 (17.3)	298 (7.2)	551 (14.2)	1030 (11.4)	
Obese	20 (1.9)	39 (0.9)	60 (1.5)	119 (1.3)	
SBP	114 ± 11.6	116 ± 11.4	118 ± 11.6	117 ± 11.7	<0.05
DBP	70.4 ± 8.55	70.6 ± 8.84	72.0 ± 8.97	71.2 ± 8.89	<0.05
HDP					
No	1008 (96.2)	3991 (96.7)	3621 (93.3)	8620 (95.2)	<0.05
Yes	40 (3.8)	136 (3.3)	262 (6.7)	438 (4.8)	
Gestational hypertension					
No	1024 (97.7)	4036 (97.8)	3732 (96.1)	8792 (97.1)	<0.05
Yes	24 (2.3)	91 (2.2)	151 (3.9)	266 (2.9)	
Preeclampsia					
No	1032 (98.5)	4082 (98.9)	3772 (97.1)	8886 (98.1)	<0.05
Yes	16 (1.5)	45 (1.1)	111 (2.9)	172 (1.9)	
GDM					
No	622 (59.4)	3137 (76.0)	3281 (84.5)	7040 (77.7)	<0.05
Yes	426 (40.6)	990 (24.0)	602 (15.5)	2018 (22.3)	
Delivery mode					
Vaginal delivery	688 (65.6)	2632 (63.8)	2195 (56.5)	5515 (60.9)	<0.05
Caesarean section	360 (34.4)	1495 (36.2)	1688 (43.5)	3543 (39.1)	
Neonatal sex					
Female	526 (50.2)	1880 (45.6)	1755 (45.2)	4161 (45.9)	0.033
Male	522 (49.8)	2247 (54.4)	2128 (54.8)	4897 (54.1)	
Birthweight	2.96 ± 0.488	3.16 ± 0.439	3.32 ± 0.432	3.21 ± 0.457	<0.05
SGA	162 (15.5)	439 (10.6)	276 (7.1)	877 (9.7)	<0.05
AGA	858 (81.9)	3367 (81.6)	3059 (78.8)	7284 (80.4)	
LGA	28 (2.7)	321 (7.8)	548 (14.1)	897 (9.9)	
Gestational week	39 (38,39)	39 (38,40)	39 (38,40)	39 (38,40)	<0.05
Early/Mid-term Preterm Infants	45 (4.3)	56 (1.4)	22 (0.6)	123 (1.4)	<0.05
Late preterm infants	80 (7.6)	191 (4.6)	110 (2.8)	381 (4.2)	
Term Infants	923 (88.1)	3880 (94.0)	3751 (96.6)	8554 (94.4)	
All	1048 (11.6)	4127 (45.6)	3883 (42.9)	9058 (1.0)	

Data are expressed as means ± standard deviation, median (IQR) or frequency (proportion). GWG, gestational weight gain; BMI, body mass index; SBP, systolic blood pressure; DBP, diastolic blood pressure; HDP, hypertensive disorders of pregnancy; GDM, gestational diabetes mellitus; SGA, small for gestational age; AGA, appropriate for gestational age; LGA, large for gestational age.

### 3.2 The correlation between GWG and adverse maternal and neonatal outcomes

**Figure 1:**
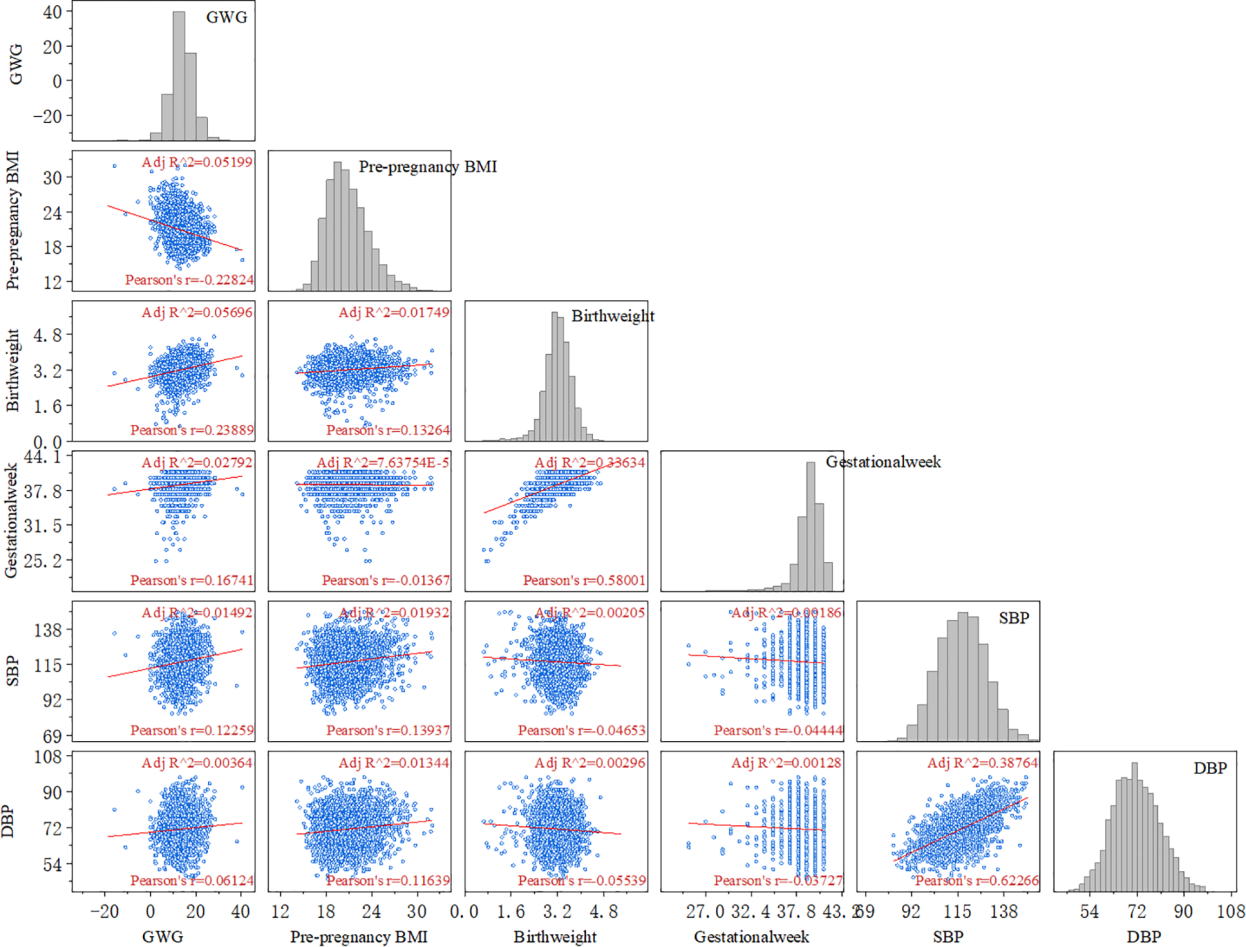
Correlation analysis between GWG and adverse maternal and infant outcomes. (GWG, gestational weight gain; BMI, body mass index; SBP, systolic blood pressure; DBP, diastolic blood pressure).

The maternal late pregnancy systolic blood pressure (r = 0.12, P < 0.05), diastolic blood pressure (r = 0.06, P < 0.05), gestational age (r = 0.17, P < 0.05), and neonatal birth weight (r = 0.24, P < 0.05) all showed a positive correlation with gestational weight gain (**Figure 1**).

### 3.3 GWG and risks of maternal and neonatal outcomes

**Figure 2:**
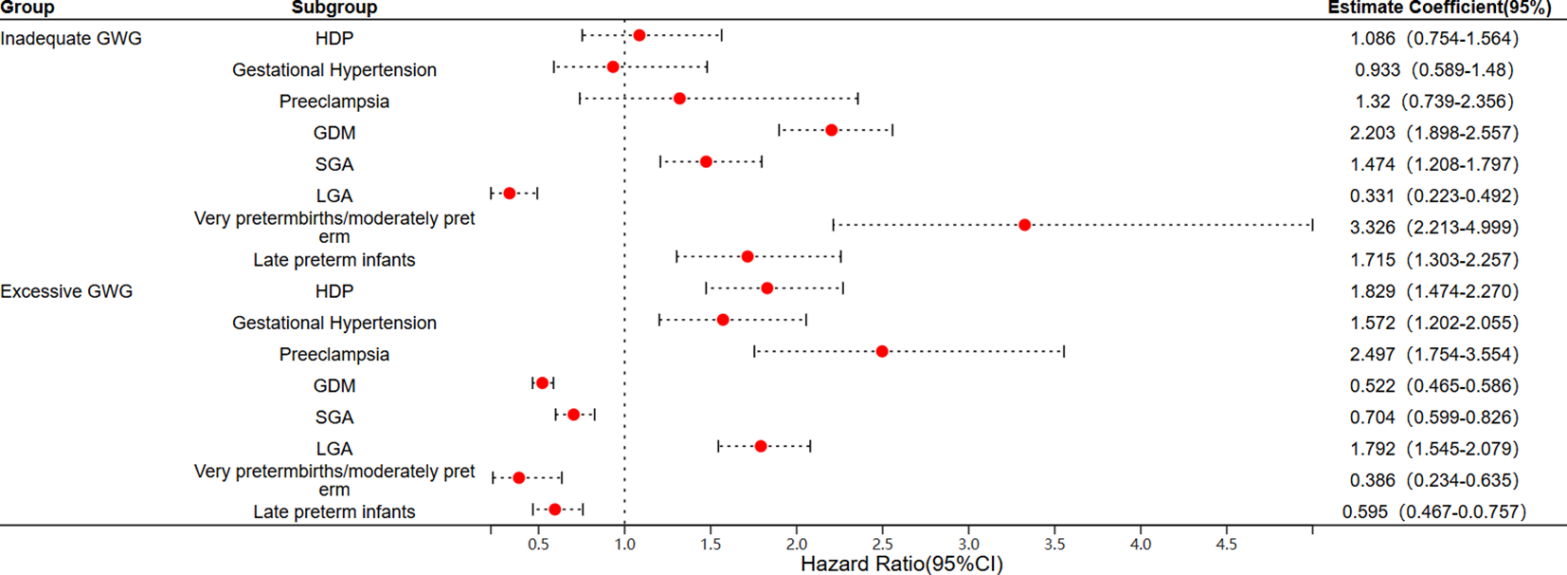
Logistic regression analysis of the risk of adverse maternal and infant outcomes associated with GWG. (GWG, gestational weight gain; HDP, hypertensive disorders of pregnancy; GDM, gestational diabetes mellitus; SGA, small for gestational age; LGA, large for gestational age).

The adjusted odds ratios (aOR) for adverse maternal and neonatal outcomes associated with insufficient or excessive GWG are presented in **Figure 2**. After adjusting for potential confounding factors such as age, pre-pregnancy BMI, and neonatal sex, the results indicated that excessive GWG is a risk factor for HDP compared to the normal weight gain group (aOR = 1.829, 95% CI: 1.474–2.270), it is associated with an increased risk of gestational hypertension (aOR = 1.572, 95% CI: 1.202–2.055) and preeclampsia (aOR = 2.497, 95% CI: 1.754–3.554). In addition, insufficient GWG increases the risk of GDM (aOR = 2.203, 95% CI: 1.898–2.557). In terms of neonatal outcomes, insufficient GWG is identified as a risk factor for SGA infants (aOR = 1.474, 95% CI: 1.208–1.797) and for both early/mid-term preterm infants (aOR = 3.326, 95% CI: 2.213–4.999) and late preterm infants (aOR = 1.715, 95% CI: 1.303–2.257). Conversely, excessive GWG is a risk factor for LGA (aOR = 1.792, 95% CI: 1.545–2.079) (**Figure 2**).

## 4 Discussion

GWG is a dynamic process, and appropriate GWG is one of the key indicators of maternal adaptation and normal fetal development. Current statistics indicated that the proportion of insufficient GWG in China ranges from 8.3% to 31.3%, while the proportion of excessive GWG is between 33.3% and 50.9%, which is slightly lower than the 60% observed in the United States (15).In this study, among the 9,058 participants, the proportion of pregnant women with insufficient GWG was 11.6%, while 42.9% experienced excessive GWG. These figures highlight that appropriate GWG remains a critical societal concern, especially with rapid economic and lifestyle changes. To meet the nutritional needs of both mother and fetus, many mothers may over-supplement, contributing to the rising percentage of excessive GWG. This study further explores the relationship between different GWG categories and adverse maternal and neonatal outcomes.

This study indicated that excessive GWG is a significant risk factor for HDP. Compared to pregnant women with normal GWG, those with excessive GWG had a substantially increased risk of developing HDP. These findings align with previous research conducted both domestically and internationally, reinforcing the importance of monitoring and managing GWG during pregnancy to mitigate associated health risks (16). The interaction between inflammation and maternal metabolic syndrome—which includes obesity, dyslipidemia, insulin resistance, and coagulation abnormalities—can lead to the early development of HDP. This process subsequently increases oxidative stress, resulting in endothelial dysfunction and inadequate maternal organ perfusion, ultimately causing HDP. During pregnancy, the body’s increased demand for fats to meet nutritional needs can lead to excessive GWG. When this results in obesity, the surplus adipose tissue may further induce the onset of HDP through these mechanisms (17). Additionally, research has indicated that patients with HDP have a significantly higher risk of experiencing cardiovascular events in the future (18). Therefore, to prevent HDP and subsequent adverse cardiovascular outcomes, it is essential to regularly monitor and manage GWG, particularly in pregnant women with pre-pregnancy BMI categorized as overweight or obese.

In our study, we found that insufficient GWG is a risk factor for GDM, while excessive GWG appears to be a protective factor. This finding is inconsistent with other research results. We speculated that this discrepancy may be due to the fact that our study only includes weight data from the third trimester, lacking weight gain data from other stages of pregnancy. Because GDM is often diagnosed in the second trimester, after diagnosing GDM, healthcare providers typically recommend weight management through a proper diet and behavioral interventions to achieve blood sugar control. Therefore, women with GDM may experience lower GWG during the third trimester. Similarly, while reviewing the literature, we also found that some studies did not identify excessive GWG as an increased risk for GDM. This may be because these studies focused on overall weight gain during the entire pregnancy, thus also including weight gained after the diagnosis of GDM. However, the total GWG of women with GDM may be influenced by interventions for GDM, and if excessive GWG is considered a potential cause of GDM, this could explain the reverse causality (19–21).

Secondly, our study observed that maternal GWG is significantly associated with neonatal birth size. Compared to the normal GWG group, excessive GWG increases the risk of LGA, while inadequate GWG is a risk factor for SGA. Research indicated that GWG above the normal range is significantly associated with an increased risk of neonatal obesity. This is due to the fact that, compared to lean women, obese women have lower insulin sensitivity. As a result, they experience heightened insulin responses during early pregnancy, which can affect early placental growth and gene expression. This leads to the release of placental factors that subsequently reduce insulin sensitivity in maternal tissues (such as skeletal muscle, liver, and adipose tissue). Ultimately, this increase in nutrient supply for fetal placental growth—coupled with excessive availability of glucose and lipids—can contribute to fetal obesity (22). Additionally, research had shown that when considering whether the association between maternal GWG and the risk of LGA is independent of pre-pregnancy BMI, the positive correlation between GWG and LGA risk remained consistent across women with different pre-pregnancy BMI categories and was independent of pre-pregnancy BMI itself (23). Similarly, the study by Tiffany et al. highlighted that, regardless of pre-pregnancy BMI, managing GWG is crucial for reducing the risks of SGA and LGA (24). These studies emphasized the public health significance of monitoring GWG, highlighting that attention to maternal GWG is crucial for influencing neonatal birth weight outcomes.

Nowadays, preterm infants were shown to have a higher risk of poor cardiopulmonary function, adverse cognitive outcomes, low blood sugar, respiratory diseases, and even increased mortality (25–29). In recent years, many researchers had recognized that GWG is negatively correlated with gestational age (30), Women with inadequate GWG have a higher risk of delivering preterm infants (31). In this study, women with inadequate GWG had a 1.7-fold higher risk of late preterm births and a 3.3-fold higher risk of early/mid preterm births compared to those with sufficient GWG. This underscored the importance of personalized GWG strategies based on pre-pregnancy BMI to prevent adverse outcomes associated with preterm births.

The strengths of this study included a sufficient sample size and the use of the 2022 Chinese recommendations for GWG as a basis for grouping. This standard was considered more applicable to pregnant women in China compared to the 2009 guidelines set by the Institute of Medicine in the United States. This relevance enhances the study’s applicability to the local population and may lead to more effective strategies for managing GWG (10). A limitation of the study was the lack of baseline data on certain factors, such as the mothers’ residence, income levels, education, dietary and exercise habits during pregnancy. This absence of information may affect the ability to fully understand the influences on GWG and related outcomes. Secondly, our study lacked weight data from various stages of pregnancy for the study population, and therefore, only the total GWG could be calculated. In addition, this study was unable to obtain other indicators that could better reflect the metabolic status of the body, so BMI was used as the measurement standard. In summary, both inadequate and excessive GWG can lead to adverse maternal and infant outcomes. Therefore, healthcare providers, including midwives and hospital staff, should focus on educating women of childbearing age about healthy GWG. Personalized advice based on pre-pregnancy BMI should be provided, along with information on the consequences of uncontrolled GWG. And it is recommended that pregnant women closely monitor their weight gain during subsequent prenatal check-ups according to the standard that specifies the recommended values for weight gain in Chinese pregnant women (9), with guidance on diet and physical activity.

## 5 Conclusion

Excessive GWG is a risk factor for the occurrence of HDP and LGA, while insufficient GWG increases the risk of GDM, SGA and preterm infants (especially early/mid-term preterm infants). Therefore, it is recommended to strengthen the monitoring of weight changes during pregnancy in women of childbearing age, guide nutritional management during pregnancy, and keep GWG within a reasonable range to prevent adverse outcomes for mothers and infants.

## Data Availability

The raw data supporting the conclusions of this article will be made available by the authors, without undue reservation.

## Supplementary material

Supplementary Figure 1 Correlation analysis between GWG and adverse maternal and infant outcomes according to the sex of newborns. **(A)** Correlation analysis when the sex of newborns is female; **(B)** Correlation analysis when the sex of newborns is male. (GWG, gestational weight gain; BMI, body mass index; SBP, systolic blood pressure; DBP, diastolic blood pressure).
