# Supplementary figures and images for: The influence of maternal gestational weight gain on adverse perinatal outcomes
Source: Front Endocrinol (Lausanne). 2025 Feb 5;16:1513344. doi: 10.3389/fendo.2025.1513344 (PMC11835695; doi:10.3389/fendo.2025.1513344)

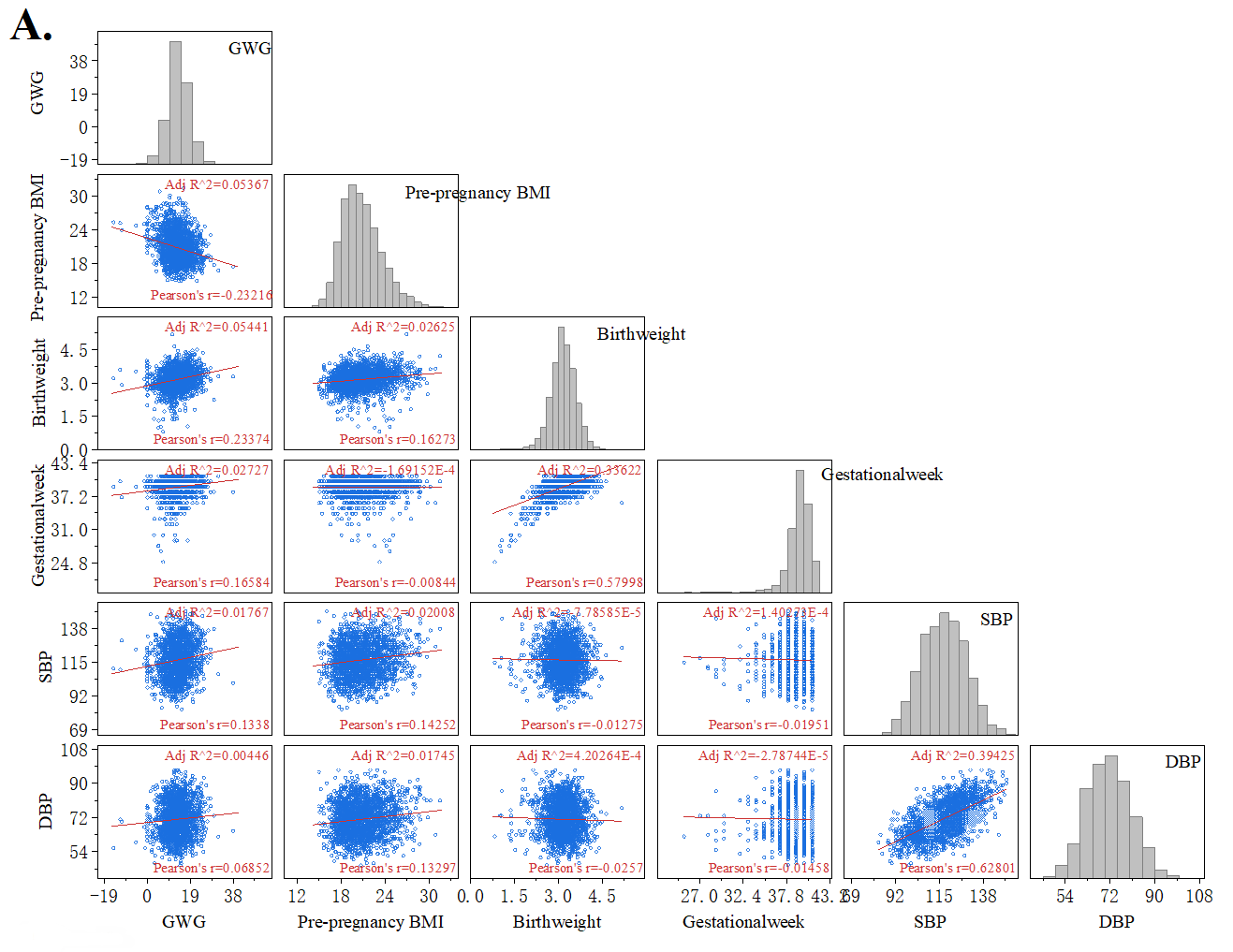

Supplement: Supplementary Figure 1 — Correlation analysis between GWG and adverse maternal and infant outcomes according to the sex of newborns. (A) Correlation analysis when the sex of newborns is female; (B) Correlation analysis when the sex of newborns is male. (GWG, gestational weight gain; BMI, body mass index; SBP, systolic blood pressure; DBP, diastolic blood pressure). [file Image1.tif]

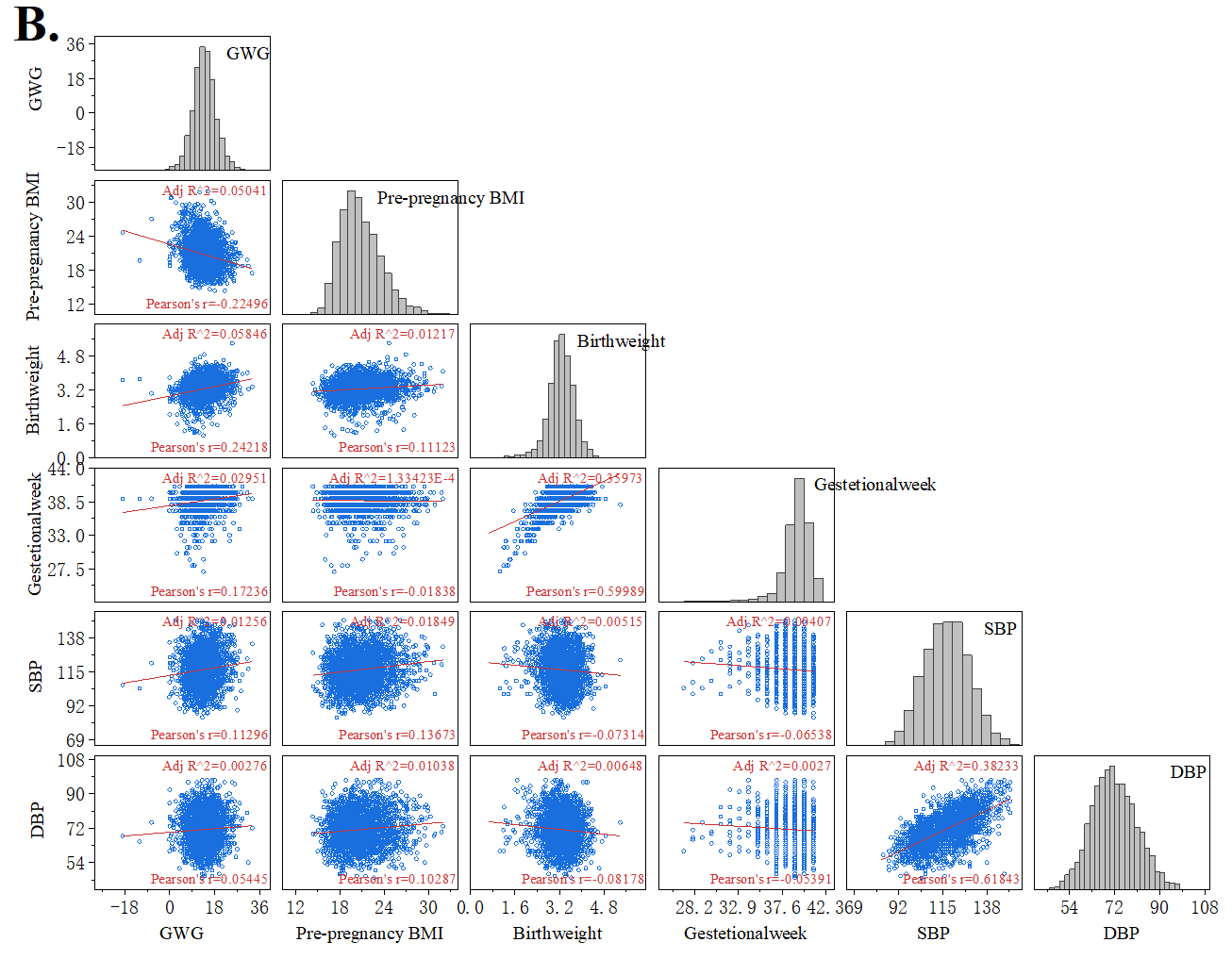

Supplement: Supplementary file 2 [file Image2.tif]
